# Genotype-protein phenotype characterization of *NOD2* and *IL23R* missense variants associated with inflammatory bowel disease: A paradigm from molecular modelling, dynamics, and docking simulations

**DOI:** 10.3389/fmed.2022.1090120

**Published:** 2023-01-10

**Authors:** Khalidah Khalid Nasser, Thoraia Shinawi

**Affiliations:** ^1^Department of Medical Laboratory Technology, Faculty of Applied Medical Sciences, King Abdulaziz University, Jeddah, Saudi Arabia; ^2^Princess Al-Jawhara Al-Brahim Center of Excellence in Research of Hereditary Disorders, King Abdulaziz University, Jeddah, Saudi Arabia; ^3^Centre for Artificial Intelligence in Precision Medicine, King Abdulaziz University, Jeddah, Saudi Arabia

**Keywords:** inflammatory diseases, IBD, genetic variants, molecular docking, protein stability, 3D modelling, MD simulation

## Abstract

Inflammatory bowel disease (IBD) is a gastrointestinal disease with an underlying contribution of genetic, microbial, environment, immunity factors. The coding region risk markers identified by IBD genome wide association studies have not been well characterized at protein phenotype level. Therefore, this study is conducted to characterize the role of *NOD2* (Arg675Trp and Gly908Arg) and *IL23R* (Gly149Arg and Arg381Gln) missense variants on the structural and functional features of corresponding proteins. Thus, we used different variant pathogenicity assays, molecular modelling, secondary structure, stability, molecular dynamics, and molecular docking analysis methods. Our findings suggest that SIFT, Polyphen, GREP++, PhyloP, SiPhy and REVEL methods are very sensitive in determining pathogenicity of *NOD2* and *IL23R* missense variants. We have also noticed that all the tested missense variants could potentially alter secondary (α-helices, β-strands, and coils) and tertiary (residue level deviations) structural features. Moreover, our molecular dynamics (MD) simulation findings have simulated that *NOD2* (Arg675Trp and Gly908Arg) and *IL23R* (Gly149Arg and Arg381Gln) variants creates rigid local structures comprising the protein flexibility and conformations. These predictions are corroborated by molecular docking results, where we noticed that *NOD2* and *IL23R* missense variants induce molecular interaction deformities with *RIPK2* and *JAK2* ligand molecules, respectively. These functional alterations could potentially alter the signal transduction pathway cascade involved in inflammation and autoimmunity. Drug library searches and findings from docking studies have identified the inhibitory effects of Tacrolimus and Celecoxib drugs on *NOD2* and *IL23R* variant forms, underlining their potential to contribute to personalized medicine for IBD. The present study supports the utilization of computational methods as primary filters (pre-*in vitro* and *in vivo*) in studying the disease potential mutations in the context of genptype-protein phenotype characteristics.

## 1. Introduction

Inflammatory bowel disease (IBD) is chronic autoimmune condition of the digestive tract (GIT) (1). Ulcerative Colitis (UC) and Crohn’s disease (CD) constitute the two main clinical forms of IBD. The specific molecular etiology of IBD is yet to be fully understood, but numerous studies show that aberrant interactions between various genetic, immunologic (e.g., mucosal immune cells) and environmental (e.g., gut microbiota) factors play a pivotal role in IBD pathogenesis (1, 2). The genetic basis of IBD is well supported by findings such as increased disease rates in monozygotic twins, and also by disease susceptibility differences among ethnic groups (3). Population genetics investigations have also revealed compelling evidence about the critical role of genetic factors in the etiopathogenesis of IBD. In recent years, the International IBD Genetics Consortium (IIBDGC), has pooled up all the GWAS findings and identified a total of 201 IBD susceptibility loci (4, 5). Among these loci, *NOD2* and *IL23R* still represent the strongest predictors for IBD susceptibility and clinical phenotypes (6–8).

*NOD2* (Nucleotide Binding Oligomerization Domain 2) is an intracellular receptor belonging to the family of cytosolic NLRs (NOD, leucine-rich repeat protein) involved in immune response by recognizing the muramyl dipeptide (MDP) component of the bacterial cell wall. *NOD2* variants like Arg70Trp, Gly908Arg, Arg702Trp and Leu1007PfsX2*NOD2* are strongly implicated in Crohn’s disease (CD) in Caucasian population (9–12). The *IL23R* gene encodes a transmembrane protein molecule belonging to type I cytokine receptor (13). This molecule initially pairs with *IL12RB1* to bind the *IL23* signaling molecule and activates JAK- STAT and NF-κB signaling pathways. This receptor is highly expressed in dendritic cells and is shown to be involved in controlling infection and chronic autoimmune diseases (14). The polymorphisms in the *IL23R* gene are also known to modulate IL23 responses and have also been reported to influence the risk of IBD development (15, 16).

Although, positive statistical associations of *NOD2* and *IL23R* genes with IBD is well known, the specific mechanisms how these genetic variants contribute to clinical phenotypes is not yet clear. It is reasonable to assume that the disease related amino acid substitution mutations cause changes in the chemical nature or position of the encoded amino acid variant, and potentially influences the bio physical characteristics (like hydrogen bonding, pH dependence and conformational dynamics) of the proteins. Although, both *in vivo* and *in vitro* studies are effective solution in this direction, but they consume lot of time and require a series of laboratory investigations. The alternate strategy for overcoming this difficulty is by predicting the specific biophysical impacts of each mutation through advanced integrated bioinformatics approaches. So many computations programs like SIFT (17), Polyphen (18), M-CAP (19), FATHMM (20), CADD (21) etc., each specializing on different prediction principles, are now available for exploring the relationship between genetic mutations and human diseases. Numerous studies have utilized these programs to screen clinically significant genetic variants in different human diseases (22–26). Therefore, in the present study, we have performed a comprehensive computational analysis of *NOD2* (Arg675Trp and Gly908Arg) and *IL23R* (Gly149Arg and Arg381Gln) variants using diverse range of machine learning approaches. The genetic sequence – protein structure relationships were studied different structural parameters like secondary structure, active sites, motifs, domains, and accessible surface areas in both wild type and mutant proteins.

Disease management strategy for IBD patients involves surgery or drug treatment, depending upon the clinical conditions and progression of inflammation (27). IBD treatment regime consists of drugs belonging to five major categories like anti-inflammatory steroids, immunosuppressive, symptomatic relief drugs, antibiotics, and biological agents. The long-term serious side effects and toxicity induction by these steroidal and non-steroidal drugs in IBD patients is seen to be unavoidable. However, this problem can be effectively minimized by screening drugs which have the potential to inhibit mutated target proteins and reduce the drug associated cellular toxicity (28). Our drug library searching revealed us that Tacrolimus and Celecoxib drugs shows specific inhibitory action on mutated forms of *NOD2* (Arg675Trp and Gly908Arg) and *IL23R* (Gly149Arg and Arg381Gln), respectively. Hence, our study provides computational evidence to repurpose Tacrolimus and Celecoxib drugs against IBD pathogenesis after conducting comprehensive *in vitro* and *in vivo* experiments.

## 2. Materials and methods

### 2.1. Variant data

The details of NOD2 and *IL23R* genes including mRNA accession number, reference number and their concerned protein sequences were retrieved from UniProt, Human Gene Mutation Database (HGMD), ClinVar, 1,000 genomes, Ensemble (and the Single Nucleotide Polymorphism Database (dbSNP). The terms like genetic mutations, genetic variations, and SNPs are used interchangeably throughout this manuscript.

### 2.2. Prediction and functional annotation of variants

dbNSFP version 2.2 was used for the functional predictions and annotations of NOD2 and *IL23R* missense mutations. The dbNSFP is a comprehensive database for functional predictions and annotations of all the potential human non-synonymous single-nucleotide variants (nsSNVs) (29, 30). The current version (dbNSFP v2.2) of the database is based on the GENCODE 9/Ensemble version 64 and it includes a total of 87,361,054 nsSNVs. The search for the nsSNVs from the database is done using a java program that executes the query in dbNSFP v2.2 on the local machine of the user. For each query it produces two prediction scores and three conservation scores along with other variant and gene annotations. In this study, we produced the prediction data for *NOD2* (Arg675Trp and Gly908Arg) and *IL23R* (Gly149Arg and Arg381Gln) genetic variants using six different algorithms e.g., SIFT, PolyPhen-2, GERP++, PhyloP, SiPhy and REVEL.

### 2.3. Structure analysis of mutations

#### 2.3.1. 3D modeling, secondary structure, and solvent accessibility methods

The structural and functional consequences of any variant can be better understood, by studying them at 3D level. Therefore, we analyzed the 3D model of selected *NOD2* (Arg675Trp and Gly908Arg) and *IL23R* (Gly149Arg and Arg381Gln) variants. The Protein Databank (PDB) does not have experimentally solved structures for *NOD2* and *IL23R*, so, we resorted to homology and/or *ab initio* based computer modeling. In this study, we used different homology modeling tools like Modeller,^1^ Swiss Model,^2^ etc., Another important computational approach used to build a protein model is, *ab initio* modeling. When an identical structure is unavailable or the target sequence has <30% identity, this approach is utilized. The I-Tasser^3^ used in the *ab initio* studies relies on the basic principle of multiple-threading alignments by LOMETS and iterative template fragment assembly simulations. The energy minimization of built protein models was done by applying the force-field of steepest descent using SPDV tool.^4^ This energy minimization step was carried out to remove the wicked contacts in a simulated protein structure. After the energy minimization step, built protein’s structural quality was assessed by Procheck^5^ tools.

The secondary structure analysis (such as helices, loops, sheets, etc.) of built models was carried out using the PDBSUM server.^6^ The active site analysis were carried out using CastP^7^ tool, this tools provide information about the active cavities, conserved amino acids and substrate binding sites present in the protein structure. Electrostatic, superpose, and solvent accessibility analysis were carried out using Pymol, Yasara,^8^ and SAS tools.^9^ The SAS analysis provides information about exposed and buried residues present in a protein, which is very crucial for comparing wild type and mutated protein models. In order to check the domains in the protein sequence, we submitted our sequence to the SangerPfam web server,^10^ which directly searches the protein sequences by expanding typical search methodology with a Pfam wrapper around the HMMER pack. The default E-value threshold used in the HMM search process was 1.0.

#### 2.3.2. Molecular dynamics (MD) simulations

The structural analysis of the *NOD2* and *IL23R* proteins was performed to evaluate the stability of wild type and variant proteins using Gromacs 4.0 and Molecular Operating Environment (MOE) softwares. The energy minimization for initial structures was performed using the steepest descent algorithm in the Gromacs 3.3 software package at a maximum of 2,000 ps time, at 300K temperature. After energy minimizing the wild type and mutated proteins, we applied restraint at 100 ps to allow solvent equilibration (NVT, NPT) around the protein. Finally full MDS was performed on all structures (wild-type and mutant models) at 20,000 ps, separately embedded in a box (box volume > 756.12 nm^3^), containing pre-equilibrated water molecules. The van der Waals interaction and particle Mesh Ewald (PME) for long range electrostatic interactions was set to >10 Å. The space between the edge of the box and protein was set at >10 Å. Episodic frontier environments were smeared in all ways. Charged ions were positioned to exchange water molecules in alternate positions, thus building the entire neutral system. The lengths of hydrogen-atom bonds were constrained using the LINCS parameters technique, at a 0.002 ps time step. For every 1 ps, the structures from the dynamic trajectory were saved. The xmgrace analysis package in GROMACS software, was used to perform all the post-dynamic studies of the trajectories (31).

### 2.4. Genetic interaction networks analysis

The protein association partners of *NOD2* and *IL23R* were studied using GeneMania tool.^11^ These databases provide data about protein association based on multiple categories of information, including physical co-occurrences, genomic neighborhood, conserved co-expression, and gene fusion, and these studies are limited to experimentally validated interactions. The input format consists of providing the query gene list. The output is a network of functional relationships for query gene and predicted related genes in the form of nodes and edges. Nodes represent genes and links represent networks. Genes can be linked by more than one type of network.

### 2.5. Protein-drug interaction analysis by molecular drug docking

At first, the potential therapeutic molecules showing an cut-off interaction score of >0.03 against *NOD2* and *IL23R* genes were identified in Drug–Gene Interaction database (DGIdb) (32). Then molecular docking analysis was performed to elucidate the functional interaction deformities of wild and mutant proteins with the query drugs. AutoDock 4.0, which is based on the Lamarckian Genetic Algorithm, is used to run docking queries for drugs and target proteins. During the docking process, the torsion angles of flexible ligands were identified by 10 independent runs. The protein structures were initially neutralized by removing ions and charges (on histidine), before applying gigaster charges to them. The grid maps were constructed around protein-ligand molecules using Autogrid module of Auto dock software program. The default parameters used in constructing the grid were 60, 60, 60 points in x, y, and z directions, a center spacing of a grid is 0.367A° (approx. 1/4 of the length of c–c covalent bond). Then, the docking parameter file was prepared with AutoDockTools (ADT). When LGA was set to 150 runs, the other default parameters were 150 conformations, population size is 50, and energy evaluations is 25,00,000. For docking parameters, the initial translation was set to 0.2A Å; the torsion to 0.5*^o^*, the quaternion to 5.0*^o^*; and the RMS cluster tolerance to 0.75 Å. The ligands that showed the most promising binding energy were chosen from the protein-ligand docking complex at the end of the docking process. Pymol-0.98 was used to analyze the resulting docking complexes.

## 3. Results

### 3.1. Pathogenic characterization of IBD variants

The SIFT and PolyPhen-2 predictions, have attributed the deleterious effect to *NOD2* (Arg675Trp and Gly908Arg) and *IL23R* (Gly149Arg and Arg381Gln). The other predictions like GERP++, PhyloP, SiPhy and REVEL scores (GERP++ RS > 0; Phylop > 0; SiPhy > 0, REVEL < 0.5) have also confirmed that these 4 SNPs affect the nucleotide sequences, which are under the high evolutionary significance (Table 1).

**TABLE 1:** Pathogenicity prediction of coding region mutations using different algorithm.

rsID	ChrPos	Gene	Amino acid substitution	Functional predictions		Evolutionary conservations prediction			Concordance tool
				SIFT^1^	PolyPhen^2^	GREP++^3^	PhyloP^3^	SiPhy^3^	REVEL^4^
rs2066844	16:50712015	NOD2	Arg675Trp	0.01	0.72	5.74	0.742	6.913	0.55051
rs2066845	16:50722629	NOD2	Gly908Arg	0.01	1	5.91	0.871	6.9139	0.705
rs76418789	1:67182913	IL23R	Gly149Arg	0	0.991	5.24	−0.432	6.0229	0.653
rs11209026	1:67240275	IL23R	Arg381Gln	0.02	0.776	5.19	0.024	7.5165	0.449

^1^SIFT < 0.05 the corresponding SNP is “Damaging”; otherwise, it is predicted as “Tolerated.”

^2^PolyPhen-2: > 0.5 prediction, “deleterious” and < 0.5, “neutral.”

^3^GERP++, PhyloP and Siphy: the larger the score, the more conserved the site.

^4^REVEL < 0.5 is neutral, >0.5 is deleterious.

### 3.2. Protein structural impact analysis of IBD variants

Structural annotations Workflow of current study represented in Supplementary Figure 1.

#### 3.2.1. 3D modeling

**FIGURE 1:**
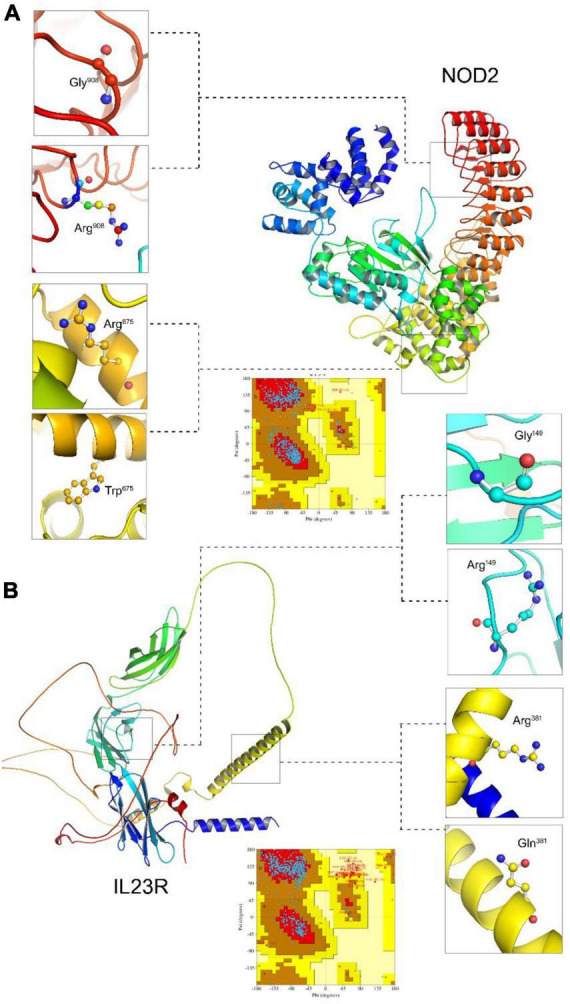
Molecular visualization of protein models (NOD2 and IL23R) and Ramachandran plots. **(A)** The NOD2 protein structure, zoom view represent localization of Gly908 (wildtype), Arg908 (mutated). **(B)** The IL23R protein structure zoom view represent localization of Gly149 (wildtype), Arg149 (mutated), Arg381 (wildtype), Gln381 (mutated).

Due to unavailability of *NOD2* and *IL23R* crystal statures in Protein Databank, we performed the BLASTp search in protein databank to check the homologous proteins with 45% identity. However, we could not find any homologues protein structures in PDB at the required threshold value. Therefore, to develop *NOD2* and *IL23R* wild type protein models, we resorted to *ab initio* based modeling approach using I-Tasser web server. The resultant output was 5 protein models for *NOD2* and *IL23R*, each. The best model was selected based on its c-scores (ranging from −5 to + 2). The top *NOD2* protein model (Figure 1A) had a c-score of −1.23 and *IL23R* had a score of −2.2 (Figure 1B). Both *NOD2* and *IL23R* were cured by an energy minimization step to remove all the bad contacts in the protein structure. *NOD2*’s energy was minimized at 2,335 fs, and the released energy was −3,25,428 KJ/Mol. For *IL23R*, energy minimization was done at 3,245 fs, and it resulted in the release of −2,3545 KJ/mol of energy. These models were further evaluated for protein quality using PROCHECK software. The *NOD2* protein model revealed that 97% of residues are in the allowed region and only 3% of residues are present in the disallowed region. For *IL23R*, 96.8% of residues are in the allowed region and 3.2% of residues are in the disallowed region of the protein.

The native *NOD2* and *IL23R* protein structures were further used as templates to create mutant protein versions using MODELLER9v3 and Swiss Model server software. All the 100 models (output from MODELLER9v3) generated per each mutant category, were further subjected to energy minimization followed by PROCHECK validation. The mutant model (Gly908Arg and Arg675Trp) of *NOD2* contains 95.2% residues in allowed regions and 4.8% in disallowed regions. The two mutant models (Gly149Arg, Arg381Gln) of *IL23R* consist of 94.2 and 96.8% of residues in the allowed region, whereas 5.8 and 3.2% of disallowed regions, respectively.

#### 3.2.2. Super positioning of native and mutant models

We compared wild and mutant protein models of *NOD2* and *IL23R* to examine their structural drifts induced by amino acid substitutions. The c-alpha backbone of the root mean square deviation (RMSD) between wild type and mutated models (Arg675Trp and Gly908Arg) of *NOD2* was found to be of 0.04 and 0.06 Å suggesting a limited potential of these mutations in inducing whole structure level alterations, respectively. However, at the amino acid residue level, these deviation was seen to be very high, i.e., 2.45 and 1.78 Å, respectively. The *IL23R* superposed on two mutated models, the C-alpha and backbone RMSDs were 0.048 and 0.052Å, suggesting limited potential of Gly149Arg and Arg381Gln mutations in inducing whole structure level alterations. Similarly, even at amino acid residue level, the deviation was minimal, that is, 1.6 and 1.48 Å (Table 2).

**TABLE 2:** RMSD values and H-bond interaction of mutant and wild type models of *NOD2* and *IL23R*.

Protein	Mutated residue	RMSD (Å)		H-Bonds
		Protein level	Residue level	
NOD2	Arg675	–	–	7 H-bods, Arg-675, Val590, Ala589, Arg678, Leu672, Ser591
	Trp675 (M)	0.04	2.453	3-Hobonds Val671, Leu672, Ala679
	Gly908	–	–	2 H-Bonds with Asn880 and Val935
	Arg908 (M)	0.06	1.78	1 H–Bond with Val935
IL23R	Gly149	–	–	–
	Arg149 (M)	0.0479	1.6	1 H-bond with Glu130
	Arg381	–	–	5 H-bonds with Ser379, Thr382, Gly383
	Gln 381 (M)	0.052	1.48	1 H-bond with Ser379

#### 3.2.3. Secondary structural annotations of IBD variants

We sought to examine the structural and functional consequences of amino acid substitutions in *NOD2* and *IL23R* proteins through diverse approaches like secondary structure analysis, and clefts analysis.

#### 3.2.4. Secondary structural features, clefts, and active site analysis of NOD2

**FIGURE 2:**
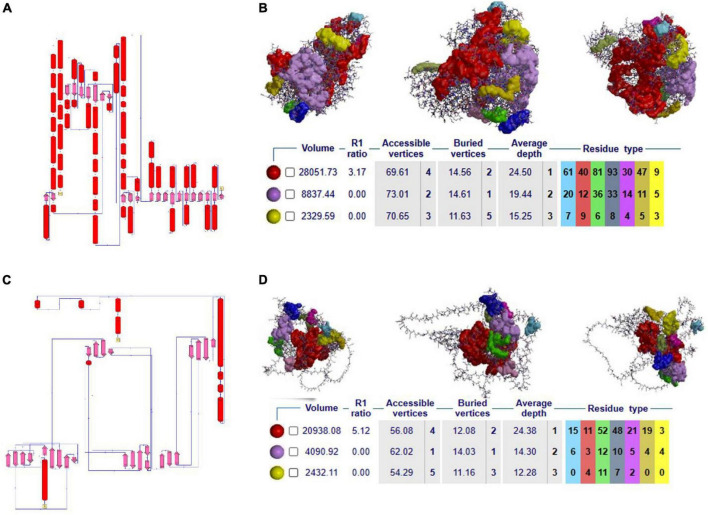
**(A–C)** 2D secondary structural conformation of NOD2 and IL23R. **(B–D)** The NOD2 and IL23R clefts in the structure, shown here as solid surfaces colored according to volume, with the largest shown in red.

Secondary structure analysis is crucial to understanding the hierarchical classification of protein structures and their polypeptide folding nature. The secondary structure of *NOD2* consists of different elements like 3 beta sheets, 12 beta-alpha-beta motifs, 2 beta hairpins, 1 beta bulge, 20 strands, 44 helices, 78 helix-helix interfaces, 68 beta turns, and 9 gamma turns. As *NOD2* is a transmembrane protein, it is made up of many helices as well as beta turns to maintain the polypeptide folding, which is important for maintaining its globular shape (Figure 2A).

Clefts are defined as gap regions existing in any protein molecule. The size of cleft often determines how protein interacts with their ligand molecules. Most of the active sites in proteins contain both deep and large clefts. The *NOD2* protein contains 4 clefts greater than 1,000 Å, out of which deepest and largest cleft located in between signal recognition and oligomerization regions is 12,085.03 Å in size. This large cleft is made up of 201 residues and consists of 72.13% accessible vertices and 13.77% buried vertices (Figure 2B).

*NOD2* ligand binding site prediction using PDBSUM showed that ADP interacts with His 603, Ser306, Thr239, Gly302, Thr240, Thr253, Thr307, Gly304 and Lys305 amino acid residue of *NOD2*.

#### 3.2.5. Secondary structural features, clefts, and active site analysis of IL23R

The *IL23R* protein consists of three regions, i.e., the C-terminal signal recognition, transmembrane and cytosolic c-terminal regions. The secondary structural features of this protein are made up of 10 sheets, 7 beta hairpins, 3 beta bulges, 37 strands, 4 helices, 80 beta turns, 40 gamma turns, and one disulfide bridge. The odd secondary structural features of *IL23R* are characterized by a low number of helices and a high ratio of turns, which further helps to maintain the stability of *IL23R* in the membrane (Figure 2C).

The *IL23R* contains 4 clefts that are larger than 1,000 Å in size. Out of these, the fourth cleft made up of 91 residues is the deepest and largest, is 6,021Å in size, and it contains 65.91% accessible vertices and 11.59 buried vertices (Figure 2D).

*IL23R* active site prediction using the CASTp server revealed the existence of two different active or ligand-binding sites in between extracellular and intracellular regions. In the extracellular region, the active site acid amino acid residues are as follows, Tyr100, Gln110, Asp118, Leu210, and Arg227. In the intracellular region, Phe530, Asn542, Glu570, Aln587, and Gly599 are predicted as active site residues.

#### 3.2.6. Solvent accessible surface area analysis of IBD variants

**FIGURE 3:**
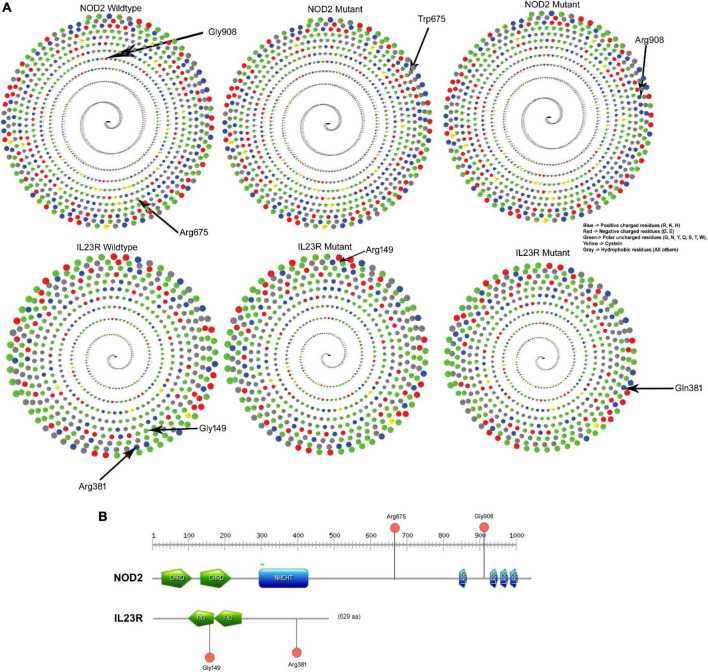
**(A)** Solvent accessibility surface area of wildtype and mutant NOD2 and IL23R. **(B)** Domain region in NOD2 and IL23R.

The native Arginine at 675th position interacts is in buried condition with more than 30% surface accessible area to solvents but the variant Tryptophan is found in exposed condition and decreases the solvent accessibility. The glycine (native) amino acid at the 908th position of the *NOD2* protein is in buried position and portrays 20% surface accessible area to solvents, whereas the substitution of arginine amino acid, due to its physical conformation, portrays 80% of the surface accessible area to solvents. The *IL23R* Phe149 and Arg381 amino acid (native) residues showed 80% surface accessible regions, with only Arg381 showing a significant shift (80–100%) in its solvent accessibility ability (Figure 3A).

### 3.3. Stability predictions of IBD variants

Any amino acid substitution is likely to affect the stability of protein structures. Therefore, to understand the structural consequences of Gly908Arg of *NOD2* and Gly149Arg and Arg381Gln of *IL23R* on their protein stabilities, we assessed their free energy changes through the DUET web server. Table 3 reveals that Gly908Arg of *NOD2* and Gly149Arg and Arg381Gln of *IL23R* mutations are destabilizing to protein stability in terms of free energy changes.

**TABLE 3:** Protein stability predictions for mutated and wild models of IL23R and NOD2.

Protein	Mutation	Stability predictions			
		mCSM*	DUET^#^	SDM^$^	Consequence
NOD2	Arg675Trp	−0.689	−0.33	−0.774	Destabilizing
	Gly908Arg	−0.895 Kcal/mol	−0.861 Kcal/mol	−0.77	Destabilizing
IL23R	Gly149Arg	−0.567 Kcal/mol	−0.304 Kcal/mol	−1.89	Destabilizing
	Arg381Gln	−0.058 Kcal/mol	−0.355 Kcal/mol	−0.5 Kcal/mol	Destabilizing

*mCSM: <-0 = destabilizing; >0 stabilizing.

^#^DUET = <-0 = destabilizing; >0 stabilizing.

^$^SDM: <-0 = destabilizing; >0 stabilizing.

### 3.4. Functional domain analysis of IBD variants

The *NOD2*, Arg675Trp variant is located 68 amino acids downstream from the winged helix domain located from 545th to 597th residues, whereas the Gly908Arg variant is in leucine rich domain 4 spanning between 897th and 1,004th amino acids. The *IL23R*, Gly149 Arg is located in Fibronectin domain 1 (129–217), whereas Arg381Gln variant lies 63 residues downstream to Fibronectin domain 2 (219–318) of the protein (Figure 3B).

### 3.5. MD simulation findings of IBD variants

The MD analysis was performed to better understand the stability of proteins in both wild and mutant states during the molecular simulation phase. We have also tried to predict physical disturbances in mutant proteins, in terms of their values corresponding to RMSD of C-alpha, radius of gyration (Rg) and solvent accessible surface area (SASA) at a 10ns solvent simulation period. The native energy minimized structures of *NOD2* and *IL23R* were used as references to compute the RMSD values of their mutant forms.

**FIGURE 4:**
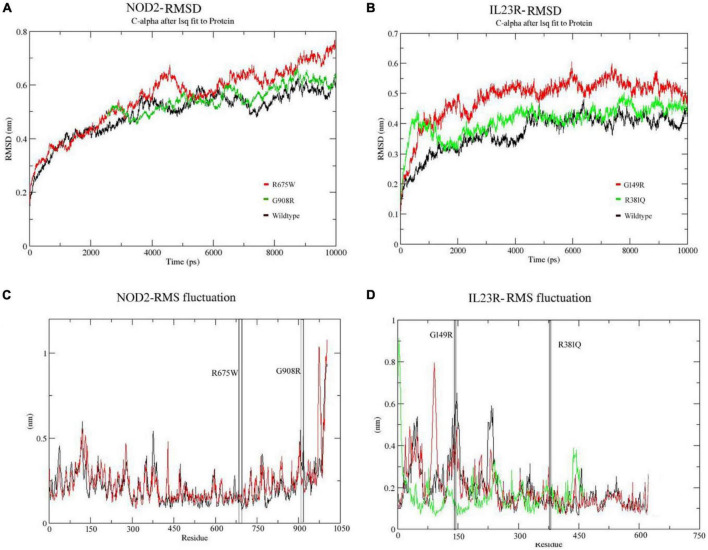
**(A,B)** Molecular dynamics RMSD of NOD2 and IL23R at 100ns. **(C,D)** MD simulation RMSF of NOD2 and IL23R at 100 ns.

In the case of *NOD2*, the molecular stability in wild type protein was achieved at 3,000 ps (0.58 nm value) over the total 10 ns simulation test period. For Arg675Trp and Gly908Arg variants, the RMSD values increased sharply after 4,000 ps and stabilized after 6,000 ps, where they achieved RMSD values in the range of 0.55 to 0.75 nm (Figure 4A). For the *IL23R* wild type, stability in the graph was achieved after 4,300 ps at a RMSD value of 0.43 nm. For Gly149Arg and Arg381Gln of *IL23R* models, a change in stability was observed at 430 ps (RMSD value is 0.39 nm) and 4,000 ps (RMSD value is 0.45 nm) (Figure 4B). In addition to this, we have also assessed the radius of gyration (Rg) and solvent accessible surface area (SASA) analyses to determine the tertiary structural features of proteins. The SASA identified the marginal exposure of Arg675Trp and Gly908Arg of *NOD2* and Gly149Arg and Arg381Gln of *IL23R* to solvent accessible areas (both hydrophilic and hydrophobic) in both native and mutant forms. However, they were found to be stable in the simulation phase. Our radius of gyration analysis showed that Rg values are different between *NOD2* wild (Rg value is 0.35 nm) and mutant (Rg value is 0.28 nm) types, suggesting the mutation induces conformational changes in the protein. The root mean square fluctuations analysis with *NOD2* and *IL23R* variants revealed flexible regions in the proteins’ 3D structures. The ligand recognition region in Gly908 (wild type) *NOD2* is more flexible (RMSF score, which is 0.6 nm) than in 908Arg (mutant), which is more rigid (RMSF score is 0.32 nm). However, this change was not able to alter the overall domain flexibility but only the flexibility of surrounding amino acid residues (Figure 4C). For *IL23R*, the wild type model showed the fluctuations or flexibility of amino acid residues in the immunoglobulin like domain (60–80 amino acids) with a value higher than 0.7 nm. The 149 Arg mutant form (RMSF value of 0.45 nm) is located in the immunoglobulin region and affects the fluctuation nature of this region. The Arg381Gln mutation of *IL23R* is located near the immune globulin like domain, and its RMSF values showed more or less similar distribution in both native and mutant forms (Figure 4D).

We have also examined the secondary structural element features of both native and mutant *NOD2* and *IL23R* models during the simulation period. At 10 ns simulation time, the wild type *NOD2* conformation had 150–256 H-bonds, while the mutant (Gly908Arg) conformation had 173–252 H-bonds. The *NOD2* mutated model showed some distinct features of secondary structural elements, which suggests that the concerned amino acid residue disturbs its natural bonding with neighboring amino acids in the polypeptide chain. At 10ns simulation period, *IL23R*’s native conformation showed 185–196 H-bonds, while the mutants *IL23R* (Gly149Arg, Arg381Gln) showed fewer H-bonds that is ∼130–145 and ∼145–168 respectively. For *IL23R*, interestingly, both the two mutated models showed similar secondary structural elements compared to their wild type counterparts. So, it is clear that changes in the amino acid sequences of *NOD2* and *IL23R* genes affect the protein’s structural stability.

#### 3.5.1. Gene interaction network findings

**FIGURE 5:**
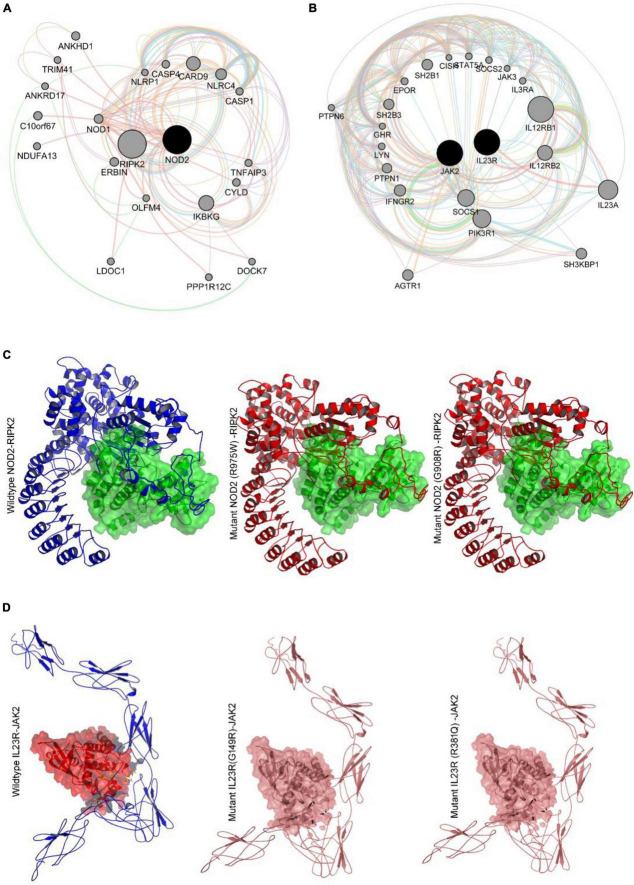
**(A,B)** Genemania protein interaction network of NOD2 and IL23R. **(C)** Molecular docking intearaction of wildtype and mutant NOD2 with RIPK2. **(D)** Molecular docking interaction of wildytype and mutant IL23R with JAK2.

Gene network analysis of *NOD2* and *IL23R* was performed with GeneMania to better understand their interacting gene partners. Figure 5A shows the physical interactions, co-expression, predicted interactions, pathways shared, co-localization, and shared protein domains network of *NOD2*. *NOD2* showed physical interaction with 18 genes, which play a very important role in many immune related pathways. *NOD2* showed co-expression with 3 genes, i.e., *RIPK2, TLR2* and *CARD9*. Interestingly, the *NOD2* interacting genes like *RIBK2*, *IKBG*, and *NKB1* are seen to share the nucleotide-binding domain leucine rich repeat receptor singling pathway, innate immune response pathway, intracellular signaling pathway, and inflammatory response pathways. Co-localization network analysis showed the interaction of *CASP4* and *TLR2* genes with *NOD2*.

*NOD2 is* also seen to share Leucine Rich Repeat and CARD Domain Containing 2 domains with *CASP1, CASP4, CASP12, CARD8, CARD9, NLRP1, NLRP4* and *RIPK2* genes. Out of all the genes involved in network, 7 genes i.e., *IKBKG, NLRC4, NFKB1, CARD9, RIPK2, XIAP* and *TLR2* plays important role in mediating the innate immune reactions. The other candidate gene *IL23R* shows direct physical interaction with IL23A and IL12RB1 genes in a network. The *IL23R* is co-expressed with IL18 and shares similar pathways with 19 genes. The gene partners which showed physical interaction, co-expression, and shared common pathways with *IL23R* gene, were all majorly involved in T-cell regulation function (Figure 5B).

#### 3.5.2. Protein-protein docking studies

Based on our gene-gene network analysis, we predicted that *RIPK2* is the best interacting partner of *NOD2*, owing to its highest confidence score (0.999) (Figure 5C). Experimental studies have proved that in the presence of ligand peptidoglycan, *NOD2* interacts with *RIPK2* to perform different intracellular reactions. Therefore, we have employed advanced docking approaches to better understand the molecular interactions between *RIPK2* and *NOD2* (both wild and mutant types). The docking analysis showed that *RIPK2* interacts with wild type *NOD2* near to its signal recognition site and interacts with Trp93, Asp113, Lys118, Leu167, Tyr192, Asn276 and releases the energy of −467.8 Kj/Mol. The Arg675Trp and Gly908Arg mutant forms of *NOD2* interacts with *RIPK2* at few similar sites to that of the wild type, but they form H-bonds with different amino acid residues and releases the energy of >-400 Kj/Mol (Figure 5C). The network analysis of *IL23R* revealed that *JAK2*, is its strong interacting partner owing to its confidence score, i.e., 0.998. Our molecular docking analysis showed that *JAK2* interacts with *IL23R*, near C-terminal region amino acid residues Trp307, Asn405, Tyr476, Gln465 and Pro 478 and releases the binding energy of −635.6 KJ/Mol. The mutant models of *IL23R* (Trp307, His345, Phe441, Asp479, Leu310, Thr472) are shown to bind the similar cleft of *JAK2* as the wild type does and release the energy of −659.4 KJ/Mol and −652.5 KJ/Mol (Table 4 and Figure 5D).

**TABLE 4:** Hex docking interaction scores of NOD2 and IL23R, wildtype and mutant models with their interaction partners.

Protein	Interaction partner	Hex binding energy (Kcal/Mol)	Difference in binding energy (Kcal/Mol)	Amino acid in interaction
NOD2 WILDTYPE	RIPK2	−467.8	–	Trp93, Asp113, Lys118, Leu167, Tyr192, Asn276
NOD2 mutant (R675W)		−418.2	49.6	Trp93, Lys118
NOD2 mutant (G908R)		−452.6	−15.2	Trp93, Asn94, Leu130, Asn276
IL23R wildtype	JAK2	−635.6	–	Trp307, Asn405, Tyr476, Gln465 and Pro 478
IL23R mutant (G149R)		−659.4	+23.8	Trp307, His345, Phe441, Asp479
IL23R mutant (R381Q)		−652.5	+16.9	Leu310,His 345, Thr472, Asp479

#### 3.5.3. Identification of potential drugs against *NOD2* and *IL23R variants*

**FIGURE 6:**
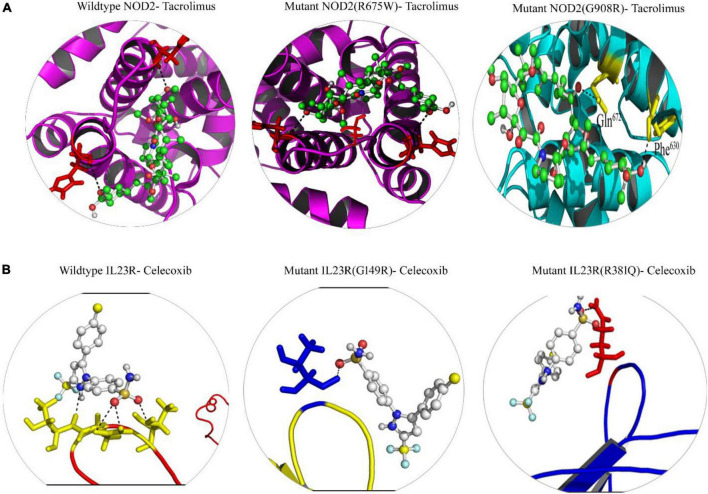
**(A)** Molecular docking interaction of wild and mutant NOD2 with drug Tacrolimus. **(B)** Molecular docking interaction of wild and mutant Il23R with drug Celecoxib.

From the gene-drug interaction database^12^ and from literature sources, we identified Tacrolimus and Celecoxib drugs which show specificity toward *NOD2* and *IL23R*, respectively. Through the advanced molecular docking approaches, we identified that Tacrolimus interacts with both wild and mutant *NOD2* at the ligand binding sites (His720 and His734 amino acids) and releases the energy of −6.12K.Cal/Mol. However, the *NOD2* mutant forms (R675W and G908R) interact with Tacrolimus drug at the same ligand binding region and releases −7.21 K.cal/mol and −6.78 K.cal/mol energy, respectively. The hex docking analysis on the ligand muramyl peptide and *NOD2* interaction revealed that muramyl peptide binds more strongly to the mutant form (with an energy release of −68.2 K.Cal/Mol) compared to the wild (with an energy release of −62.5 K.Cal/Mol) form of *NOD2* (Figure 6A). For *IL23R*, both wild and mutant protein forms showed greater interaction with the Celecoxib drug, although their interacting poses are different. The Celecoxib interacts with the Thr261 amino acid residue in wild *IL23R* and releases the energy of −3.25 K.Cal/Mol. However, the same drug showed the highest interaction (in the form of H-bonds) with Thr261, Asn262 and Thr264 amino acid residues of mutant *IL23R* (G149R) with a binding energy of −10.42K.Cal/Mol. The second *IL23R* mutant (R381N) showed an interaction with Gln263 amino acid residue and released the energy of −4.89 K.Cal/Mol (Figure 6B and Table 5).

**TABLE 5:** Docking energies of Drugs vs. NOD2 and IL23R (wild/mutant).

Protein	DRUG	Cluster^a^	RMSD^b^	Binding energy^c^ (Kcal/mol)	Inhibition constant^d^ (*Ki*)	No of H bonds	Amino acid involved in interaction
NOD2 wild type	Tacrolimus	38	0.458	−6.12	32μM	2	His720 and His734
NOD2 mutant (R675W)	Tacrolimus	18	1.43	−7.21	15 μM	2	Gln672, His720 and Phe630
NOD2 mutant (G908R)	Tacrolimus	32	1.256	−6.78	10 μM	2	Gln672 and Phe630
IL23R wildtype	Celecoxib	40	0.125	−3.25	53 μM	1	Thr261
IL23R mutant (G149R)	Celecoxib	44	0.225	−10.42	21.879nM	3	Thr261, Asn262 and Thr264
IL23R mutant (R381N)	Celecoxib	25	1.32	−4.89	42 μM	1	Gln263

^a^Indicative of the total number of binding modes produced.

^b^Heavy atoms root-mean-square deviation with respect to the experimental structure.

^c^The change in binding free energy is related to the inhibition constant using the equation: Δ*G = RT* in *Ki*, where R is the gas constant 1.987 cal K-1 mol-1, and T is the absolute temperature assumed to be 298.15 K.

^d^Estimated inhibition constant at 298.15 K.

## 4. Discussion

The experimental elucidation of the genotype-protein phenotype relationship is an uphill task owing to the number of variant discoveries being added to the already existing huge IBD mutation data (33). In this context, computational prediction algorithms act as reliable tools for prioritizing candidate genetic mutations based on the nature of their impact (negative, neutral, or positive mutations) on proteins. In the current investigation, we have systemically applied diverse computational strategies to characterize the IBD variants based on their evolutionary constraints on coding regions. These strategies included algorithmic screening of genetic mutations based on the nucleotide sequence and protein structure conservation (integrated support vector machine learning algorithms) (ex: SIFT, PloyPhen2, GERP++, PhyloP and SiPhy) across different mammalian species (34). The rationality behind using multiple prediction methods is to generate consensual variant predictions.

The importance of comprehensive computational predictions of missense variants in CA2, LDLRAP1 and SQSTM1 genes has been well demonstrated (24, 35, 36). In the recent study, Polyphen-2, when compared with SIFT, M-CAP and CADD tools, can make better pathogenicity predictions for familial hypercholesterolemia (FH) causative LDLRAP1 mutations (24). Further verification of different computational tools like SIFT, PolyPhen, M-CAP, CADD in screening PCSK9 missense mutations causative to FH is also well demonstrated (37). Few other studies have also asserted the usefulness of various computational algorithms in predicting the damaging ability of nucleotide sequence variations belonging to human disease related genes (34, 38, 39). The quantitative measurement of each constrained element in GERP++ is according to the magnitude of the substitution deficit, measured as “rejected substitutions” (RS). Here, the negative and positive RS scores are inversely proportional to evolutionary selections, where in negative scores often are often considered to be strong signal of biological function. From our GERP++ analysis, we discovered that all the four SNPs fall in evolutionarily conserved regions (RS < 0) and are under strong negative selection. But, due to inherent differences of coding region with regards to the pattern of evolutionary constraints, analysis of population specific genetic variations in regulatory regions, which undergoes evolutionary remodeling, will be of greater assistance to better understand the human specific evolutionary selections (8).

The specific structural and functional implications of any genetic mutation (on its corresponding protein) can be predicted based on the information about the significance of amino acids it alter. In this context, amino acid residues which fall in evolutionarily conserved regions serves as important pointers in understanding the clear effects of genetic mutations of human diseases. Highly pathogenic mutations in a protein hotspot or active region may disrupt the activity of the protein (40). Additionally, studying the mutations at 3 dimensional structure level will help us in understanding the specific structural deformities a particular amino acid variant is likely to inflict on protein. The mapping of the mutation onto three-dimensional protein structures and analyzing these changes at the structural level will help to find the exact point where they loss function/alter interactions with proteins (41). As of today, the tertiary structural conformation of native and/or mutant *NOD2* (Arg675Trp, Gly908Arg) and *IL23R* (Gly149Arg, Arg381Gln) is not yet resolved through laboratory experimental x-ray crystallographic or NMR spectroscopic methods. So, we built the 3 dimensional structural models of *NOD2* (Arg675Trp, Gly908Arg) and *IL23R* (Gly149Arg, Arg381Gln) proteins by *ab initio* method, and analyzed for its biophysical characteristics like stability differences, structural deviations, solvent accessible surface areas and secondary structural features such as polypeptide folding (42).

The structural divergence of core proteins often correlates with amino acid sequence divergence in an exponential function manner (43). In our structural deviation analysis, the Arg675Trp and Gly908Arg mutations of NOD2 have indicated their significance by causing huge structural drift at amino acid residues but not at whole structure level deviations. The NOD2 mutation Arg675Trp variant is not directly localized in the domain region of the protein. However, the Arg675 amino acids form an H-bond network with the surrounding amino acids. Whereas in the mutated condition, the H-bond network is depleted, and this might cause structural alteration in the NOD2 protein (44). The second mutation G908R of NOD2 is actually located in Lucine Rich Domain (735–1,040a.a) (LRRD), which folds as horseshoe enabled by its rich content of hydrophobic amino acid leucine (45). Although, this domain is not directly involved in protein-protein interacting sites, but it assist in stabilizing the NOD2 polypeptide folds which have active site domains (46). Thus, it is explicable that Gly908Arg mutation is capable of altering the NOD2 interaction ability by changing its H-bond properties. In contrast, Gly149Arg and Arg381Gln mutations of IL23R are not seen to inflict any significant structural deviations at both amino acid and whole structure level. Single compared to multiple amino acid residue substitutions often fails to invoke compensatory effects (caused in case of multiple amino acid substitutions) on protein structure, they induce changes in side chain charge (47), active site conformations and polypeptide complementarity, which are essential for maintaining the protein structures. The two mutations (G149R and R381Q) of *IL23R* are located in extracellular domain and C-terminal cytoplasmic portions, respectively (48). Due to mutation G149 in *IL23R* structure the transmembrane domain the first beta barrel of *IL23R* increases its size from Ser251-Lys258 to Val251-Lys258; this structural change may alter the binding ability of extracellular domain of *IL23R* with its ligand (49). In the second mutated protein structure of *IL23R* (R381Q), helical structure (Leu468-Thr472) is converted into loop component in the extracellular domain portion, there by altering its binding ability with first intermediate molecules critical for inducing cascade of intracellular cellular signaling mechanisms underlying inflammatory bowel disease.

We used the molecular dynamics simulation approach to examine the natural and mutant NOD2 and IL23R structures at the atomic level to gain a better understanding about missense mutations induced impacts on protein structures. From the simulation trajectory values, basic metrics such as RMSD, RMSF, hydrogen bond numbers, and SASA were evaluated. Molecular stability and flexibility changes were estimated from RMSD and RMSF values. Stability is the fundamental property enhancing biomolecular function, activity, and regulation. In our study, the distinct change in the RMSD trajectories of mutated forms of *NOD2* and *IL23R*, indicate the differences in the route of alteration of structures from the starting conformation to their final states despite the initial structures being identical. This evidence clearly states the impact of amino acid substitutions on the dynamics of the proteins. The RMSF data also showed the mutated regions are highly flexible in both proteins (*NOD2* and *IL23R*) mutations state. A clear insight of stability loss was observed in both RMSD and RMSF parameters, which is further given the evidence by decreasing the number intermolecular hydrogen bonds in mutant structures. Intermolecular H-bonds are most important factors in maintain the protein conformation and creates stable interaction between the protein and its binding partner (50).

The exponential function between structural divergence of protein and amino acid sequence variation is variable based on the mutation rates of amino acid residues, which occupies either buried or accessible positions on protein surface (34). Following this principle, we identified that both Arg 675 (native) and 908 glycine (native) amino acids of *NOD2* protein is in buried position and portrays only 20, 40% surface accessible area to solvents, whereas the substitution of tryptophan and arginine amino acids, due to its physical conformation, portrays 80 and 60% surface accessible area to solvents. The Phe149 and Arg381 amino acid (native) residues of *IL23R* showed 80% surface accessible regions, out of which, only Arg381 showed the major drift (80–100%) in its solvent accessibility ability. An explanation in accordance with our observation is that amino acid residues in core portion of proteins is differentially conserved in terms of their sequence and structure, than those that solvent accessible (51). The good correlation of solvent accessibility and stability analysis suggests that *NOD2* and *IL23R*, further confirms that drift in solvent accessibility affects the protein stability.

The networking analysis of genes is a useful approach to understand the functional interactions and associated signaling cascades. The networks shown in form of arcs (relationships) and nodes (entity) are based up on their connectivity levels with other interacting proteins. The *NOD2* networking analysis suggested its strong role in immune mediated pathways. The *NOD2* showed physical interaction with 18 genes, which are playing very important role in many immune related pathways. The *NOD2* showed co-expression with 3 genes i.e., *RIPK2*, TLR2 and CARD9. Interestingly, the *NOD2* interacting genes like RIBK2, IKBG and NKB1 are seen to be sharing Nucleotide-binding domain leucine rich repeat receptor singling pathway, Innate immune response pathway, Intracellular signaling pathway, Inflammatory response pathways. Co-localization network analysis showed the interaction of CASP4 and TLR2 genes with *NOD2. NOD2* is also seen to share Leucine Rich Repeat And CARD Domain Containing 2 domains with CASP1, CASP4, CASP12, CARD8, CARD9, NLRP1, NLRP4 and *RIPK2* genes. Out of all the genes involved in network, 7 genes i.e., IKBKG, NLRC4, NFKB1, CARD9, *RIPK2*, XIAP and TLR2 plays important role in mediating the innate immune reactions. The genetic network *NOD2* showed that *RIPK2* is its highest interaction partner owingto the confidence string score of 0.999. *RIPK2* plays an important role in modulation of immune response (both adaptive and innate). The exposure of peptidoglycan content of foreign particles can activate both *NOD2* and NOD1, which further interacts with *RIPK2* through two caspase recruitment domains (CARD-CARD) leading to the tyrosine phosphorylation and activation of NF-Kappa B (52). Once NFKB is released and translocates into nucleus it activates hundreds of genes responsible for immune responses, growth control and apoptotic mechanisms. To better understand the interactions between *NOD2* with *RIPK2*, protein-protein docking study was performed, where we identified that *RIPK2* binds at CARD domain (95–182AA) of *NOD2* (45). The NOD2 mutant form Arg675Trp forms weaker interactions with RIPK2 compared to wildtype conditions, indicating the mutation may destabilize the interaction of RIPK2 with NOD2 protein. The second mutant condition (G908R) state, *NOD2* interacts with *RIPK2* at the same CARD domain. However, the mutant *NOD2* (G908R, located in LRRD domain) shows differential binding conformation in terms of interacting amino acids, leading to energy differences between native and mutant forms *NOD2* protein against *RIPK2.*

Our multidimensional computation strategy (pathogenic prediction of nucleotide sequence variations in addition to molecular dynamics simulations) confirms that the R675W and G908R, mutation alters the structural conformation of *NOD2*, thus interaction with *RIPK2* and eventually dysregulate the *NOD2* −*RIPK2* signaling pathway. There have been several reports, which indicated the link of *NOD2* mutations with aberrant immune responses in terms of temporal and quantitative effects of activation of the TLR2-*NOD2* −*RIPK2* pathway on secretion of IL-10 further disturbing the between pro- and anti- inflammatory responses against gram-positive bacteria (53).

The other candidate gene *IL23R* shows direct physical interaction with IL23A and IL12RB1 genes in a network. The *IL23R* is co-expressed with IL18 and shares similar pathways with 19 genes. The gene partners showed physical interaction, co-expression and shared common pathway with *IL23R* gene are all majorly involved in T-cell regulation function (54). The selection of *JAK2*, best interacting partner of *IL23R* was based on the confidence string score of 0.093. Janus tyrosine kinase 2 (*JAK2*), a non-receptor type, class III protein is the intermediate molecule that binds to *IL23R*, whose activation by IL23, phosphorylates STAT and activates NFKB pathway that is essential for stimulating inflammatory reactions involving T-cells, NK cells and possibly certain macrophage/myeloid cells. Owing to the lack of data on *IL23R* and *JAK2* molecular binding characteristics, we performed *IL23R*-*JAK2* molecular docking. It was found that *JAK2* interacts with the cytosolic terminal of native *IL23R* (at Trp307, Asn405, Tyr476, Gln465 and Pro 478 amino acid residues). Interestingly, even in mutant state the *IL23R* also shows the samelevel interaction with *JAK2* but its binding affinity (+16.9 and +23.8 Kcal/Mol) is decreased when compared to wild type *IL23R* and *JAK2* conformation. A recent study G149R mutation of *IL23R*, observed the reduced expression of STAT3 (48). Cellular functional assays have also observed that R381Q mutation affects the constitutive association of *JAK2* with *IL23R*, with effects on subsequent STAT3 recruitment, phosphorylation, and transcriptional activation (55).

As of today, no specific drug or drug targets are established for treating IBD, except steroid medications, which just reduces the severity of inflammatory reactions in IBD patients (56). From our multidimensional computational approach, we propose that, *NOD2* and *IL23R* have the potential to act as molecular targets. The drug, Tacrolimus interacts with *NOD2* at the ligand binding site of *NOD2* and may positively upregulate different crucial pathways involved in immune suppressive mechanisms. On the other hand, Celecoxib, a non-steroidal anti-inflammatory drug shows strong interaction with mutant *IL23R* compared to its wild type, there by regulates the *IL23R* function. Our computational findings pave the way to test non-steroidal anti-inflammatory bowel disease drugs in experimental conditions.

In conclusion, our study found that SIFT, PolyPhen-2, GERP++, PhyloP, SiPhy and REVEL computational algorithms are very helpful in analyzing *NOD2* (R675W and G908R) and *IL23R* (G149R and R381N) variants. The secondary structure, tertiary structure, and stability prediction approaches have demonstrated how the loss-of-function variants induce minor structural drifts, shift free energy values, and reduce the conformation flexibility of the *NOD2* and *IL23R* protein molecules. Overall, our comprehensive computational approach adds a layer to estimate the deleterious potential of genetic variants associated with IBD. This study recommends implementing multidimensional genotype – protein phenotype assessment methods as a pre-laboratory approach in developing personalized medicine for IBD patients carrying NOD2 (R675W and G908R) and IL23R (G149R and R381N) variants.

## Data availability statement

The datasets presented in this study can be found in online repositories. The names of the repository/repositories and accession number(s) can be found in this article/Supplementary material.

## Author contributions

KN and TS: conceptualization, data curation, formal analysis, methodology, supervision, visualization, and writing original draft and editing. KN: funding acquisition, project administration, software, and validation. Both authors contributed to the article and approved the submitted version.

## Footnotes

1
https://salilab.org/modeller/

2
https://swissmodel.expasy.org/

3
https://zhanggroup.org/I-TASSER/

4
https://spdbv.unil.ch/

5
https://saves.mbi.ucla.edu/

6
http://www.ebi.ac.uk/pdbsum

7
http://sts.bioe.uic.edu/castp/index.html

8
www.yasara.org

9
www.abren.net/asaview

10
www.ebi.ac.uk/interpro/

11
https://genemania.org/

12
http://dgidb.genome.wustl.edu/
