# Supplementary figures and images for: Genotype-protein phenotype characterization of NOD2 and IL23R missense variants associated with inflammatory bowel disease: A paradigm from molecular modelling, dynamics, and docking simulations
Source: Front Med (Lausanne). 2023 Jan 10;9:1090120. doi: 10.3389/fmed.2022.1090120 (PMC9871552; doi:10.3389/fmed.2022.1090120)

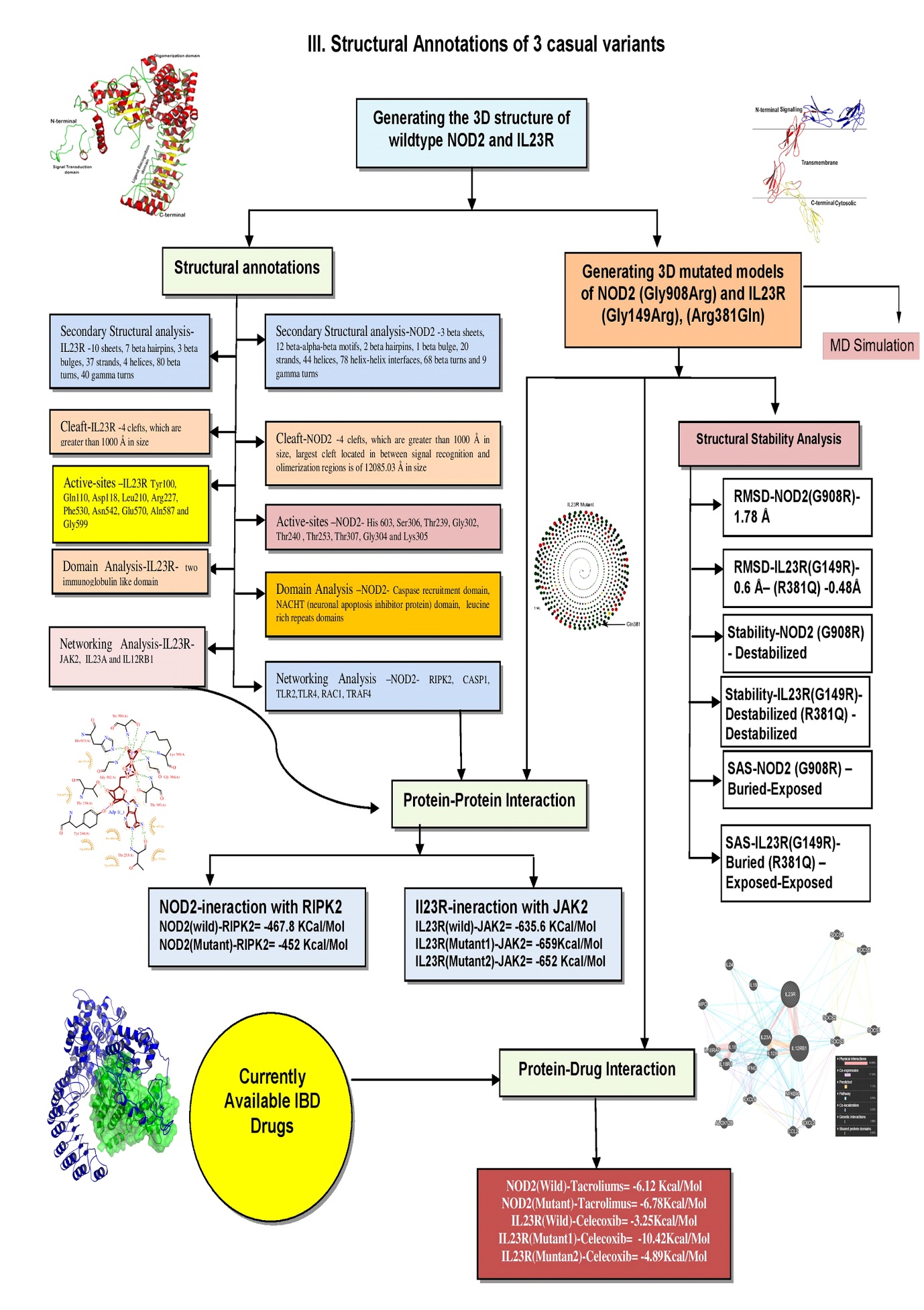


Supplementary Figure 1: Flow of current analysis on IBD NOD2 and IL23R

Supplement: Supplementary file 1 [file Table_1.DOCX]
